# Short term physician visits and medication prescriptions for allergic disease associated with seasonal tree, grass, and weed pollen exposure across the United States

**DOI:** 10.1186/s12940-021-00766-3

**Published:** 2021-07-21

**Authors:** Shubhayu Saha, Ambarish Vaidyanathan, Fiona Lo, Claudia Brown, Jeremy J. Hess

**Affiliations:** 1 Centers for Disease Control and Prevention, National Center for Environmental Health, 4770 Buford Hwy, GA 30341 Atlanta, USA; 2 Department of Atmospheric Sciences, College of the Environment, University of Washington, Seattle, WA USA; 3 Department of Emergency Medicine, School of Medicine, University of Washington, Seattle, WA USA; 4 Department of Environmental and Occupational Health Sciences, School of Public Health, University of Washington, Seattle, WA USA; 5 Department of Global Health, Schools of Medicine and Public Health, University of Washington, Seattle, WA USA

**Keywords:** Pollen, Rhinitis, Allergy medication, Pollen alert, Health risk assessment

## Abstract

**Background:**

While year-round exposure to pollen is linked to a large burden of allergic diseases, location-specific risk information on pollen types and allergy outcomes are limited. We characterize the relationship between acute exposure to tree, grass and weed pollen taxa and two allergy outcomes (allergic rhinitis physician visit and prescription allergy medication fill) across 28 metropolitan statistical areas (MSA) in the United States.

**Methods:**

We obtained daily pollen data from National Allergy Bureau (NAB) monitors at these 28 MSAs for 2008–2015. We revised the NAB guidelines to classify taxa-specific pollen severity each day. Daily information on allergic rhinitis and prescribed allergy medications for individuals with employer-based health insurance from the IBM MarketScan Research database for these MSAs. We combined the daily pollen and health data for each MSA into a longitudinal dataset. We conducted a MSA-specific conditional quasi-Poisson regression analysis to assess how different levels of pollen concentration impact the health outcomes, controlling for local air pollution, meteorology and Influenza-like illness (ILI). We used a random effects meta-analysis to produce an overall risk estimate for each pollen type and health outcome.

**Results:**

The seasonal distribution of pollen taxa and associated health impacts varied across the MSAs. Relative risk of allergic rhinitis visits increased as concentrations increased for all pollen types; relative risk of medication fills increased for tree and weed pollen only. We observed an increase in health risk even on days with moderate levels of pollen concentration. 7-day average concentration of pollen had stronger association with the health outcomes compared to the same-day measure. Controlling for air pollution and ILI had little impact on effect estimates.

**Conclusion:**

This analysis expands the catalogue of associations between different pollen taxa and allergy-related outcomes across multiple MSAs. The effect estimates we present can be used to project the burden of allergic disease in specific locations in the future as well inform patients with allergies on impending pollen exposure.

**Supplementary Information:**

The online version contains supplementary material available at 10.1186/s12940-021-00766-3.

## Introduction

Allergies related to pollen exposure are widespread and occur throughout the year in many locations. While the global burden of disease related to aeroallergens is elusive, prevalence of allergic rhinitis, the most common form of pollenosis, is estimated to be approximately 20% [1] and pollen allergy prevalence has increased significantly in recent years [2, 3]. Pollen seasons in the northern hemisphere have lengthened as a result of anthropogenic climate change [4], and worsening disease burdens have been attributed to climate change in certain regions [5, 6]. Several studies have projected substantial increases in aeroallergen exposure and associated burden of disease in the US [7] and Europe [8], and there is concern that other atmospheric changes in the Anthropocene will amplify the impacts of increased pollen exposure [9].

Sensitivity to pollen tends to develop after pollen exposure early in life [10] and sensitization at a young age is strongly predictive of symptoms during childhood and later in life [11]. Sensitized individuals commonly have allergies to multiple types of plant pollens [12]. A relatively small number of wind-pollinated plants, including approximately ten tree taxa [13, 14] as well as several grasses [15] and weeds [16], appear to be responsible for the majority of pollenosis. Short-term exposure to allergenic pollens immediately drives symptomatic disease in sensitized individuals [17] and can result in health complications several days after exposure [18].

Direct medical cost related to allergic rhinitis in the United States is estimated to be around 3.4 billion dollars, with prescription medications being the largest component [19]. It is common to manage pollen allergies with over-the-counter (OTC) medications [20] and OTC medication purchases are commonly used as indicators of allergic disease [13, 21]. Approximately 80% of allergic rhinitis symptoms can be managed successfully with inhaled corticosteroids and other medications [20]. However, patients with moderate to severe symptoms seek medical care – 12.3% in the US in one study [22] – and receive prescription medications [23]. Thus, medication use and physician visits are important health indicators to track allergic disease exacerbation. Emergency department (ED) visits for allergic rhinitis are relatively rare [22], unlike for patients with allergic asthma that has been linked to increase in exposure to pollen [13, 24].

Prior assessments of adverse health impacts associated with pollen have several limitations. Many are limited by geography, focusing only on few specific locations [13, 25], inclusion of a few pollen types [13, 24], or examine a limited number of health outcomes [21]. The objective of our study is to broaden the characterization of the relationship between acute exposure to a wide range of allergenic pollens prevalent in the continental United States and health outcomes related to allergic rhinitis and allergy medication fills. We use the existing National Allergy Bureau (NAB) guidelines as a benchmark to inform risk communication efforts across the pollen seasons and multiple locations. We control for other potential exposures like air pollution [13], and influenza-like illness (ILI) known to cause upper respiratory symptoms like rhinitis (https://www.cdc.gov/flu/professionals/acip/clinical.htm).

## Methods

### Pollen data

We obtained daily pollen data for 2008–2015 from 28 monitors that are part of the NAB network of the American Academy of Allergy, Asthma, and Immunology (AAAAI) (See Supplemental figure SF1). We geocoded the physical address of these locations to identify the Metropolitan Statistical Area (MSA) in which these monitors reside. The selection of these monitoring stations was based on our assessment of completeness of the pollen data as well as availability of the health data for those specific locations. The monitors were all located in urban areas.

*Identification of pollen season start and end dates:* We use a novel approach to determine the start and end of the pollen season for each monitor [26]. For taxa with annual total counts above 2,000 pollen grain*day/m^3^, we define the start date as the day when the cumulative sum over that pollen year reaches a threshold of 50 pollen grain*day/m^3^. For taxa with lower annual total counts, we define the start date as the date on which cumulative sum reached a threshold of 2.5% of the historical mean total count, with the intent of creating a functionally equivalent threshold. The start date of the pollen season is computed for each pollen taxa at each station location for every year, and days with missing data do not contribute. The end date is calculated in a similar manner to the start date. For taxa with high mean total counts (> 2,000 pollen grain*day/m^3^), the end date is defined as the date at which the cumulative sum from that date to the end of the pollen year is less than 50 pollen grain*day/m^3^. If the historical mean total count is low, then the end date threshold is calculated as the date at which accumulated pollen concentration reaches 97.5% of the historical mean total count for the season. The overall pollen season is defined as the earliest start date and the latest end date for any taxa in a year for each monitor. While the reported pollen data vary substantially by taxa across stations, the common taxa that were included in the tree, grass and weed pollen are available in the study used to define the pollen season [26].

*Pollen exposure characterization:* We used the pollen thresholds published by the NAB as the benchmark (https://www.aaaai.org/global/nab-pollen-counts/reading-the-charts) to characterize pollen concentrations each day. These guidelines are currently widely used to communicate health risks from pollen exposure. Since a scan of the literature revealed a wide range of pollen levels deemed adverse to health [27–29], we modified the NAB thresholds to examine health effects at relatively low levels of pollen instead of solely focusing on days with extremely high concentrations [26]. For each type of pollen, we defined five categories as ‘low’, ‘moderate’, ‘moderately high’, ‘high’ and ‘very high’ based on the following concentration ranges (by grain*day/m^3^—(tree pollen: < 15, 15–90, 90–250, 250–1500, > 1500; grass pollen: < 4, 4–19, 19–50, 50–100, > 100; weed pollen: < 9, 9–50, 50–100, 100–250, > 250) (Supplemental table ST1 for more details). NAB uses a broad range of pollen concentrations to define the ‘high’ category. We broke it into two categories – ‘moderately high’ and ‘high’. For grass and weed, we also lowered the NAB threshold to define ‘very high’ as few locations reported grass and weed pollen at such extremely high levels. We linearly interpolated pollen data by tree, grass, and weed within their specific seasons each year to account for missing data when pollen was not measured (e.g. most stations do not measure pollen over the weekends). We used the imputed pollen concentrations to classify each day in the study period into the five pollen categories for tree, grass and weed respectively. This was the main pollen exposure variable of interest. We conducted a series of sensitivity analyses to compare the results with percentile-based definition of pollen concentration and consecutive days of exposure. We calculated the 25^th^, 50^th^ and 75^th^ percentile values for tree, grass and weed pollen based on the daily distribution from 2008–2015 for each site [30, 31]. Each day was then assigned to one of the four percentile-based categories with values between 0-25^th^ percentile being ‘low’ and above 75^th^ percentile being ‘high’. We compare the effect estimates using same day pollen concentrations with those averaged over 7 days from date of the health episode to check if consistently high levels of pollen over consecutive days was associated with a higher health risk.

### Health data

For the years 2008–2015, we obtained health data from the Truven Health MarketScan® Research database (Commercial Claims and Encounters (CCAE)). The database is a large convenience sample from the US population with employer-based health insurance. The database provides information on healthcare encounters of active employees, dependents and early retirees who used a fee-for-service, capitated or partially capitated health insurance plan. While the coverage varies across years, the database provides healthcare information for around 40 million individuals each year. The large sample size provides information on healthcare use across different parts of US. The database captures de-identified patient-level healthcare outcomes with age, gender, date of healthcare service, MSA of patient residence and a list of diagnoses codes based on the ninth revision of the International Classification of Diseases (ICD-9-CM). Patients were included in the study with either (i) a fill of prescribed allergy medications related to steroids and antihistamines (see Supplemental table ST2 for details) or, (ii) a physician visit with a diagnosis of allergic rhinitis (AR) (ICD-9-CM = 477.0). We calculated a daily count of medication fills (MF) by MSA based on the day of the fill. Since many AR patients require multiple follow-up physician visits following initial symptom development from exposure to pollen, we estimated two different daily counts of AR visits based on a patient’s first visit in a calendar year, as well as all visits through the year. The results presented focuses only on the count of first physician visits during the year.

We collected data on Influenza-like Illness (ILI) from the Centers for Disease Control (CDC) Fluview website. For each of the ten Health and Human Services (HHS) Regions in the country, weekly information on an index for ILI was collected for the 2008–2015 time period.

### Environmental data

For this analysis, we created daily MSA-level estimates of environmental variables such as daily measures of temperatures, relative humidity, and air pollutants. The daily measures of maximum temperature and humidity were derived using predictions from the North American Land Data Assimilation System Phase 2 (NLDAS) model, available at 0.125 degrees (approximately 14 km × 14 km) spatial resolution [32]. The grid-level predictions were first converted to county-level estimates and then to MSA-level estimates of temperature in Fahrenheit (°F) and relative humidity (%), using a population weighted approach [33, 34].The daily measures of air pollutants, such as daily maximum 8-h average ozone concentrations in parts per billion (ppb) Ozone (O_3_), and daily 24-h average Particulate Matter (PM_2.5_) (micrograms/m^3^) were available from the Bayesian space–time Downscaler (DS) fusion modelling framework that was developed by EPA and its partners. This generates predictions of ozone and PM_2.5_ by U.S. Census tracts [35]. These tract-level air pollutant predictions were averaged, using tract populations as spatial weights, to generate MSA-level estimates of O_3_ and PM_2.5_.

### Data linkage

We identified the MSA where each of the pollen monitors were located. We linked the daily data on pollen, health and environmental variables by MSA. Based on the HHS region that each MSA belonged to, we linked the ILI information to this dataset such that each day of the week was assigned the same ILI index value.

### Statistical analyses

The dependent variables in our analyses were daily counts of allergy medication fills (MF) and physician visits with a diagnosis of allergic rhinitis (AR). We adopted a Conditional Quasi-Poisson framework to assess how these counts varied across days with different categories of pollen exposure, controlling for ‘overdispersion’ and the day of week [36] for each location. The base model contained categorical variables based on daily tree, grass, and weed pollen concentrations. Since we observed overlap in the temporal distribution of the three pollen types, we included all three pollen indicators in the same model. We compared the risk estimates for each pollen category to see how the health risks increased with increase in pollen concentrations. We sequentially added variables for ILI, air pollution and meteorological factors to check if they affected the association between pollen categories and the health outcomes. We used a random effects meta-analysis approach to produce one representative effect estimate across all locations included in the study. The meteorological and air pollution factors were included in the model as continuous variables and their associations modeled as linear. All analyses was conducted in R software (Version 4.0.3). We present the relative risk estimates from the meta analyses in the manuscript and the location-specific risk estimates for all pollen categories in the Supplemental material. To display the variation in risk estimates across locations and pollen types, we use the relative risk for ‘high’ pollen days, as only a small number of days were classified as ‘very high’ based on the pollen data we had for all locations.

We conducted a series of sensitivity analyses. For AR visits, our main outcome of interest were daily counts based on a patient’s first visit in a calendar year. As a sensitivity analysis, we also examined daily counts based on all AR visits through the pollen season. For medication fills, we divided the medications into antihistamines and steroids to compare differences in risk estimates for the two types. We conducted a sensitivity analysis based on definition of pollen exposure (comparison of modified NAB thresholds with percentile-based pollen categories), duration of exposure (comparison of same-day pollen exposure with a 7-day average), and linear interpolation of pollen data (comparison of pollen categories using the daily imputed pollen concentrations with the raw data with missing observations)..

## Results

The 28 MSAs included in the analysis are shown in Supplemental Figure SF1. In Table 1, we show the distribution of different types of pollen by location. The average length of the entire pollen season varies by location. Many stations reported missing pollen data for a large percentage of days within the study period, providing a rationale to impute the missing values and avoid reducing the statistical power of the analysis. The distribution across the ‘moderately high’, ‘high’ and ‘very high’ pollen levels varied noticeably across locations and pollen types. For example, Atlanta shows a large number of days with tree pollen, but relatively few for weed pollen and even less for grass. We observed a relatively small number of days in the ‘very high’ pollen category across the three types of pollen in most locations during the study period. The higher number of days across the ‘moderate’ to ‘high’ pollen categories were more relevant in terms of assessing the health risks from exposure to pollen across entire seasons. For stations where any of the pollen types had 5 percent or less days in the ‘high’ pollen category for the study period, we excluded that pollen type in the health risk analysis. We thus estimate risk estimates for 28 MSAs for tree pollen, 18 for grass pollen and 19 for weed pollen. The distribution of the health outcomes by gender and age groups are presented in Supplemental table ST3. The cases of MF and AR comprised of individuals from 0–65 years. For the study, we analyzed 5.6 million cases of medication fills of which 52% were for females and 13% were for children (0-17 years); 1.1 million allergic rhinitis related physician visits (first visit of the season) of which 51% were for females and 24% were for children (0–17 years).

**Table 1:** Descriptive statistics of pollen distribution from 2008–2015; Average annual season length and range based on all three types of pollen combined; ^a^ missing pollen days calculated based on days within the entire pollen season; ^b^ Total number of days over the entire study period in ‘low’ (L), ‘moderate’ (M), ‘moderately high’ (MH), ‘high’ (H) and ‘very high’ (VH) categories are based on daily pollen concentrations (grains/m^3^): (tree pollen: 90–250, 250–1500, > 1500; grass pollen: 19–50, 50–100, > 100; weed pollen: 50–100, 100–250, > 250). Table cells in grey indicate very low prevalence of the type of pollen if number of days in ‘high’ pollen category were <  = 10 days during the entire study period

Location	Average (range) annual pollen season length (days)	Missing pollen (% of days) ^a^	Number of Tree pollen days ^b^					Number of Grass pollen days ^b^					Number of Weed pollen days ^b^				
			L	M	MH	H	VH	L	M	MH	H	VH	L	M	MH	H	VH
Atlanta, GA	282 (272,302)	28	1368	334	149	132	68	1809	208	34	*.*	*.*	1723	274	43	11	*.*
Austin, TX	312 (306,327)	30	1147	358	145	196	93	1590	301	68	*10*	*6*	1704	170	44	60	.
Baltimore, MD	209 (195,229)	6	1389	163	87	122	34	1268	308	102	74	41	1572	171	44	*8*	*.*
Chicago, IL	181 (167,206)	30	740	212	123	53	6	770	266	84	14	*.*	619	409	81	24	1
College Station, TX	308 (294,317)	27	1453	271	125	190	51	1634	328	94	22	17	1612	300	81	89	13
Colorado Springs, CO	233 (224,247)	9	965	449	302	152	2	1188	518	135	23	6	1143	500	139	85	3
Dayton, OH	251 (235,285)	32	1360	237	120	173	35	1482	306	104	23	10	1609	210	47	59	.
Erie, PA	173 (165,190)	38	611	128	90	135	20	684	234	42	20	4	692	219	52	21	.
Eugene, OR	279 (224,344)	35	951	400	176	84	3	995	272	99	69	177	1571	44	.	*.*	*.*
Houston, TX	320 (314,323)	34	700	207	91	135	44	741	316	95	21	6	852	162	54	80	30
Kansas City, MO	240 (224,254)	31	868	143	106	193	75	598	424	195	81	89	780	311	82	155	60
Louisville, KY	242 (228,269)	2	2200	219	174	230	16	2449	239	109	31	11	2401	244	110	84	.
Madison, WI	186 (161,217)	46	632	111	83	107	10	789	101	48	*5*	*.*	553	222	87	80	1
Minneapolis, MN	197 (179,216)	22	662	106	81	128	38	823	137	46	*7*	*2*	616	252	88	58	1
Waterbury, CT	171 (169,182)	31	613	126	84	140	42	901	93	7	*3*	*1*	883	113	9	*.*	*.*
Oklahoma City, OK	318 (269,327)	33	1007	314	192	225	60	955	392	305	124	26	1142	389	68	188	19
Omaha, NE	231 (218,250)	4	1523	214	167	164	43	1600	364	109	29	10	1349	381	143	231	8
Rochester, NY	191 (175,211)	30	729	137	167	151	2	821	198	113	40	14	846	173	54	113	.
Saint Louis, MO	261 (240,280)	30	1488	175	114	148	65	1694	178	75	27	16	1405	392	106	87	.
Salt Lake City, UT	221 (211,236)	37	874	227	69	38	2	923	235	50	*1*	*1*	754	387	63	*6*	*.*
San Antonio, TX	306 (205,328)	9	992	446	160	151	44	995	664	157	*4*	*.*	957	608	125	126	4
San Jose, CA	321 (228,333)	17	1150	790	295	136	7	2015	240	66	30	30	2184	179	17	*2*	*.*
Seattle, WA	187 (171,210)	6	658	514	231	181	19	1233	262	98	*10*	*.*	1451	141	6	*4*	*1*
Springfield, MO	229 (224,233)	31	669	133	82	107	16	502	359	101	21	23	460	413	72	62	.
Tulsa, OK	294 (252,314)	53	802	156	119	135	52	734	270	166	65	24	877	200	37	117	32
Waco, TX	330 (328,333)	30	507	353	336	571	72	1269	232	193	112	130	1364	176	89	219	88
Washington, DC	236 (212,254)	36	1191	162	120	163	25	1365	241	47	*8*	*.*	1457	195	9	*.*	*.*
York, PA	204 (192,217)	33	801	172	114	150	19	1032	125	59	33	7	975	237	30	14	*.*

In Table 2, we provide the relative risk (RR) estimates for MF and AR associated with all pollen categories and the three pollen types from the meta analyses. We observe a dose–response relationship between the pollen categories and health outcomes for all types of pollen. While the ‘very high’ pollen category was consistently associated with the highest RR, the larger confidence interval around these estimates compared to other pollen categories were due to the small number of days classified as ‘very high’. For ‘high’ pollen days, we see a significant increase in risk of MF for tree [RR = 1.11; 95%CI = 1.09, 1.14] and weed [RR = 1.08; 95%CI = 1.06, 1.10] pollen, with grass pollen being marginally insignificant [RR = 1.03; 95%CI = 0.99, 1.07]. For ‘high’ pollen days, we observe a statistically significant increase in risk of AR for tree [RR = 1.20; 95%CI = 1.10, 1.31], grass [RR = 1.29; 95%CI = 1.15, 1.42] and weed [RR = 1.31; 95%CI = 1.15, 1.47] pollen. We observe statistically significant increase in RR of MF and AR even on days with ‘moderate’ levels of pollen. This finding is particularly important for AR outcomes, as days with ‘moderate’ pollen levels show an increase in RR of a physician visit by 7%, 17% and 12% for tree, grass and weed pollen respectively. The location specific RR for MF and AR across all five pollen categories are provided in Supplementary tables ST4 and ST5 respectively.

**Table 2:** Meta-analyzed relative risk estimates of allergy medication fills and allergic rhinitis physician visits (first visit in calendar year) on days with different categories for tree, grass and weed pollen. Same-day pollen concentrations are used to define the pollen categories. Relative risk estimates derived from models with weekly ILI index, air pollution measures (daily PM2.5 and Ozone) and meteorological factors (daily maximum temperature, total precipitation, and average wind speed) as covariates

**Allergy medication fills**									
	Tree pollen			Grass pollen			Weed pollen		
**Pollen level**	RR	95% CI		RR	95% CI		RR	95% CI	
Low	1			1			1		
Moderate	**1.04**	***1.03***	***1.05***	1.00	*0.99*	*1.01*	1.01	*0.99*	*1.02*
Moderately high	**1.06**	***1.05***	***1.08***	1.00	*0.98*	*1.02*	**1.05**	***1.03***	***1.07***
High	**1.11**	***1.09***	***1.14***	1.03	*0.99*	*1.07*	**1.08**	***1.06***	***1.10***
Very high	**1.22**	***1.16***	***1.27***	**1.07**	***1.00***	***1.14***	**1.15**	***1.12***	***1.18***
**Physician visit for allergic rhinitis**									
	Tree pollen			Grass pollen			Weed pollen		
**Pollen level**	RR	95% CI		RR	95% CI		RR	95% CI	
Low	1			1			1		
Moderate	**1.07**	***1.01***	***1.13***	**1.17**	***1.08***	***1.26***	**1.12**	***1.01***	***1.23***
Moderately high	**1.08**	***1.02***	***1.15***	**1.27**	***1.13***	***1.41***	**1.18**	***1.06***	***1.29***
High	**1.20**	***1.10***	***1.31***	**1.29**	***1.15***	***1.42***	**1.31**	***1.15***	***1.47***
Very high	**1.40**	***1.14***	***1.66***	**1.34**	***1.16***	***1.53***	**1.30**	***1.12***	***1.47***

**Fig. 1:**
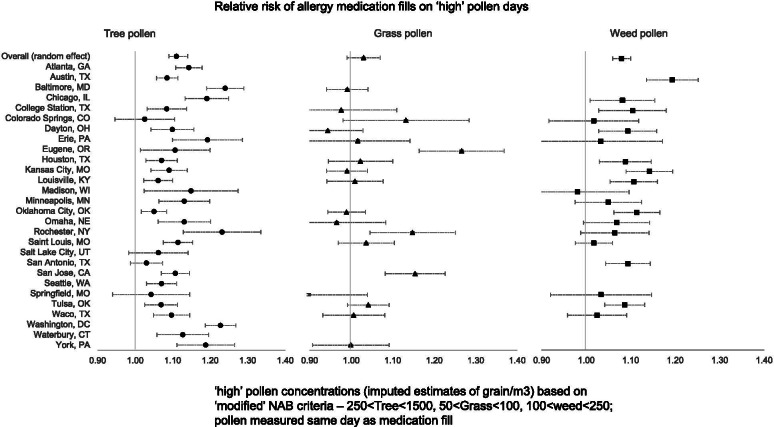
Location-specific relative risk of prescription medication fills on days when the (same day) pollen concentration is in the ‘high’ category compared to the ‘low’ category.’High’ pollen level is defined by pollen concentrations (grains/m^3^) – 250 < Tree < 1500; 50 < grass < 100, 100 < weed < 250. Relative risk estimates derived from models with weekly ILI index, air pollution measures (daily PM2.5 and Ozone) and meteorological factors (daily maximum temperature, total precipitation, and average wind speed) as covariates

We observed that the RR estimates varied by location and pollen types. In Fig. 1, we show the relative risk (RR) of MF on ‘high’ pollen days (instead of ‘very high’ pollen days which were very small in number) compared to ‘low’ pollen days. In these models, we considered same day pollen values, and included all the environmental covariates and ILI index. Besides the RR from the meta analyses that is shown at the top for comparison, the figure shows the variation in risk across locations for specific pollen types, as well as how the risk changes in each location by pollen season. We found statistical significance of the RR of MF in 24 out of 28 locations for tree pollen, 3 out of 18 locations for grass pollen, and 10 out of 19 for weed pollen. For sensitivity analyses, we examined if the RR for medication fills were different between antihistamines and steroid prescriptions. The RR for antihistamine fill on a ‘high’ tree pollen day was 1.13 [95% CI = 1.10, 1.16] compared to RR for steroid 1.09 [95% CI = 1.05, 1.14]. The differences in RR for antihistamines and steroids were similarly small for grass pollen, but the pattern was reversed for weed pollen – on ‘high’ pollen days the RR for antihistamines was 1.05 [95% CI = 1.02, 1.09] and RR for steroids was 1.11 [95% CI = 1.06, 1.15]. These results are presented in Supplemental table ST6.

**Fig. 2:**
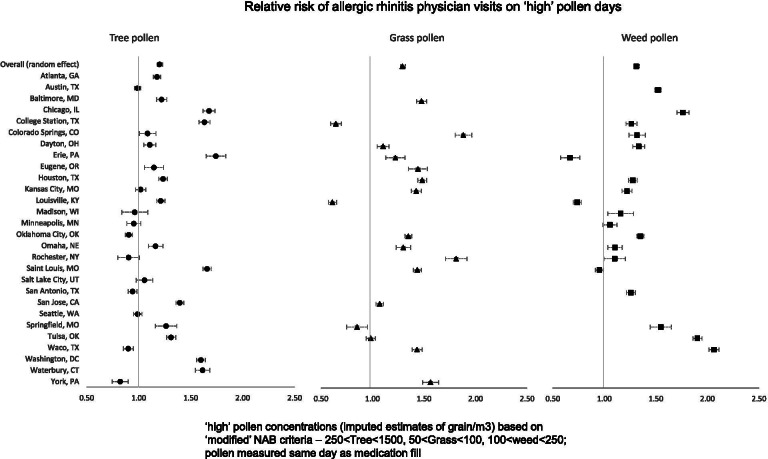
Location-specific relative risk of physician visits for Allergic Rhinitis on days when the (same day) pollen concentration is in the ‘high’ category compared to the ‘low’ category. Only the first visit during the pollen season is considered.’High’ pollen level is defined by pollen concentrations (grains/m^3^) – 250 < Tree < 1500; 50 < grass < 100, 100 < weed < 250. Relative risk estimates derived from models with weekly ILI index, air pollution measures (daily PM2.5 and Ozone) and meteorological factors (daily maximum temperature, total precipitation, and average wind speed) as covariates

In Fig. 2, we show the RR of first AR physician visit during the pollen season on ‘high’ pollen days using the model with all covariates. We found statistical significance of the RR of AR in 13 out of 28 locations for tree pollen, 11 out of 18 locations for grass pollen, and 10 out of 19 locations for weed pollen. As a sensitivity analysis, we observed much weaker associations between pollen levels and any AR visit that happened during the pollen season. For example, the RR of the first physician visit for allergic rhinitis on a ‘high’ tree pollen day was 1.20 [95% CI = 1.10, 1.31] compared to RR of all AR visits 1.10 [95% CI = 1.03, 1.18]. This pattern persisted for grass and weed pollen.

In Table 3, we show how the RR estimates are sensitive to four different measures of pollen – (i) ‘high’ pollen level based on same day imputed concentration, (ii) ‘high’ pollen level based on a 7-day average of imputed concentration, and (iii) ‘high’ pollen level based on same day non-imputed concentration, and (iv) days in the 75^th^ percentile values of the pollen distribution during the study period in each location. The RR corresponding to 7-day average pollen measure were consistently higher than the RR corresponding to the same day pollen measure. The differences were more apparent for AR – the RR associated with the 7-day average tree pollen measure was 1.31 [95% CI = 1.13, 1.49] compared to the RR associated with same day tree pollen measure of 1.20 [95%CI = 1.10, 1.31]. We observed similar patterns for grass and weed pollen as well. This indicates that persistently high levels of pollen exposure likely increase the risk of AR visits. The risk estimates for MF and AR did not differ when we compared the imputed vis-à-vis the raw daily values with missing values. We saw that RR associated with the same-day and 75^th^ percentile-based measures were similar for MF, but the latter was lower for AR.

**Table 3:** Meta-analyzed relative risk estimates of allergy medication fills and allergic rhinitis physician visits (first visit in calendar year) on days designated as – (i) ‘high’ pollen level based on same day imputed values, (ii) ‘high’ pollen based on 7-day average of imputed values; (iii) ‘high’ pollen based on same day non-imputed values of pollen (with missing pollen observations), (iv) days with pollen concentrations >=75^th^ percentile of the pollen distribution during the study period. ’High’ pollen level is defined by pollen concentrations (grains/m^3^) – 250 < Tree < 1500; 50 < grass < 100, 100 < weed < 250. Relative risk estimates derived from models with weekly ILI index, air pollution measures (daily PM2.5 and Ozone) and meteorological factors (daily maximum temperature, total precipitation, and average wind speed) as covariates

**Allergy medication fills**									
	Tree pollen			Grass pollen			Weed pollen		
**Pollen measure**	RR	95% CI		RR	95% CI		RR	95% CI	
Same day (imputed)	**1.11**	***1.09***	***1.14***	1.03	*0.99*	*1.07*	**1.08**	***1.06***	***1.10***
7 day average (imputed)	**1.16**	***1.13***	***1.18***	1.03	*0.98*	*1.08*	**1.11**	***1.08***	***1.15***
Same day (non-imputed)	**1.10**	***1.08***	***1.13***	**1.05**	***1.02***	***1.08***	**1.09**	***1.07***	***1.11***
Days in top quartile of pollen	**1.11**	***1.09***	***1.14***	1.03	*0.99*	*1.07*	**1.06**	***1.04***	***1.08***
**Physician visit for allergic rhinitis**									
	Tree pollen			Grass pollen			Weed pollen		
**Pollen measure**	RR	95% CI		RR	95% CI		RR	95% CI	
Same day (imputed)	**1.20**	***1.10***	***1.31***	**1.29**	***1.15***	***1.42***	**1.31**	***1.15***	***1.47***
7 day average (imputed)	**1.31**	***1.13***	***1.49***	**1.35**	***1.19***	***1.51***	**1.48**	***1.28***	***1.67***
Same day (non-imputed)	**1.20**	***1.10***	***1.30***	**1.33**	***1.19***	***1.47***	**1.28**	***1.12***	***1.43***
Days in top quartile of pollen	**1.14**	***1.06***	***1.23***	**1.28**	***1.16***	***1.41***	**1.24**	***1.10***	***1.38***

We did not observe any pattern in the association of the weekly ILI index, air pollution measures (daily PM2.5 and Ozone) and meteorological factors (daily maximum temperature, total precipitation, and average wind speed) to the estimated RR of MF and AR. The RR estimates were not sensitive to the inclusion of these variables in the models either. The ILI index showed inconsistent results. For the models on medication fills, it was statistically significant in only seven locations, out of which only two showed an increased risk, and there was no apparent spatial or temporal pattern that plausibly explained the observed associations. For the analysis on allergic rhinitis, it was significant in seventeen locations, out of which ten showed an increases risk, and seven showed a reduction in risk. In the models for medication refills, daily average 8-h maximum ozone concentrations increased the risk in 11 locations (at the 95% significance level). In our analyses, we observe very weak association between PM2.5 and ozone with allergic rhinitis visits. In the models for medication fills, daily average precipitation reduced the risk in only five locations (at the 95% significance level), while daily maximum temperature reduced the risk in 11 locations and increased the risk in two locations (at the 95% significance level).

## Discussion

Aeroallergen exposure is a major source of morbidity and appears to be on the rise globally as the climate warms. Developing a broad range of exposure-outcome associations, controlled for potential confounders, is an essential step in estimating disease burden associated with aeroallergen exposure. Our study adds to the literature on the health impacts of pollen by (i) examining two health outcomes –prescription medication fills and allergic rhinitis physician visits, that are relatively understudied; (ii) providing estimates of health risks for three broad categories of pollen (tree, grass, and weed), thus covering the entire pollen season; (iii) covering 28 locations across the United States; (iv) controlling for potential environmental factors (like air pollution, temperature, precipitation) and potential biases that could arise from seasonal influenza-like illness. We conduct a series of sensitivity analyses to examine how these associations vary by different constructs of pollen exposure (based on concentrations and temporal lags) and health outcomes. We observe a statistically significant increase in risk of allergic disease even at moderate levels of pollen as defined by the National Allergy Bureau. Given the high burden of allergic disease in the country, these location-specific results could inform recalibrating the risk communication strategy to issue alerts before the high pollen levels are reached.

We refrain from comparing the effect estimates from our study with other studies due to the different approaches used to measure pollen and health outcomes. Yet, it is useful to highlight a few studies that show results qualitatively similar to our study. In France, same day grass pollen count was associated to a binary indicator of severe allergic rhinitis cases [RR 1.08 95%CI = 1.01, 1.14] [15]. Studies in France [23] and Belgium [37] have found a strong association between increases in tree and grass pollen and increases in anti-allergic medication use using claims data. The risk estimates for medication fills in the Belgian study for tree and grass pollen are comparable to the estimates we derived. A study in New York City showed strong association between over-the-counter (OTC) allergy medication sales and increase in tree pollen season [13].

We found that the inclusion of the environmental variables had little impact on the magnitude of the risk estimates for medication fills and allergic rhinitis. While a study in Korea observed positive association between temperature and tree-pollen related hospital admissions [38], meteorological conditions had no impact on medication sales during the tree pollen season in New York City, NY [13] and Bridgeport, CT [39]. Studies have reported contrasting associations between OTC medications with air pollution [13, 25]. Similarly, the impact of air pollution on allergic rhinitis cases are inconclusive [15, 40–42].

There is quite a bit of variation in the lagged associations of pollen concentration and health outcomes in the literature. For example, the temporal association between high tree pollen and over-the-counter medication sales were found to last for 0–3 days in New York City [13]. The effects on allergy medication use were concentrated on the present-day tree pollens but remained significant until 3 days lag time in the case of grass pollens [23]. In our meta-analysis, we notice that the risk of the first allergic rhinitis visit of the season is strongly correlated with a 7-day average of pollen exposure for all three type of pollen, though there are variations across the locations. We do not observe such strong effects of longer lags for the risk of medication fills.

Overall, this work provides helpful new insights for risk assessment and other aspects of public health practice related to aeroallergens. Specifically, the significant increase in risk of allergic complications even on days with moderate levels of pollen concentrations suggest that risk communication should focus on early warning even at moderate exposure levels. These findings have implications for ongoing surveillance, estimations of disease exacerbation risk associated with pollen among sensitized individuals, and projections of pollen-related disease burden related to climate change, as widespread shifts in allergenic plant distribution [43] and pollen phenology [4] have been observed. The findings provide additional support for using medication fills as a surveillance indicator for allergic rhinitis, the management of which is principally via outpatient ambulatory care [23]. This could also facilitate surveillance of allergy symptoms in regions without pollen observations. The findings also have implications for land use and climate change adaptation and mitigation activities that include widespread tree planting to reduce urban heat islands and promote carbon fixation [44], which can inadvertently result in increased exposure to allergenic pollens [45], allowing for more comprehensive health impact assessment and comprehensive synthesis of environmental information in health protection [46].

The study has some limitations to note. Assignment of pollen data from a single monitoring location to health data obtained for those living in a large metropolitan area raises concerns for exposure misclassification. Different instruments (Rotorod and Burkard) and manual inspections to count pollen could introduce unknown biases in the measurements that we are unable to control for. The pollen monitors are located in urban areas that prevented us from estimating the health risks in rural areas. The health dataset only contains information on individuals who have employer-based health insurance that limits the generalizability of the results to the wider population. The health data only provides information on medication fills and not how individuals used the medication. As a result, we could not assess how medication use was associated with increase in pollen exposure. Location-specific information of the specific plant species that contribute to relatively high levels of pollen and thus to increase in allergic complications would be most useful for designing pollen early warning systems to provide timely information for susceptible individuals. Future analysis will focus on the identification of the most allergenic species in these locations and consider a wider range of respiratory health impacts. Another topic of interest will be to assess potential variation in the impact of pollen exposure on individuals with different levels of susceptibility to pollen.

Our results also suggest several additional avenues for further exploration. The relationship between pollen exposure and incidence of sensitization has been characterized for relatively few populations and is important to estimating overall disease burden related to allergic disease. The question of whether certain species within the general categories of trees, weeds, and grasses may be driving the observed relationships also remains unanswered. Lastly, while there are several markers of morbidity associated with pollen allergy, there are as yet no standardized estimates of disability such as disability adjusted life year estimates to associate with the disease, complicating standardized estimates of disease burden in the US and elsewhere. As pollen exposure, sensitization, and related allergic disease burden grow, these questions are likely to become increasingly pressing.

## Conclusion

Aeroallergens are a significant source of morbidity worldwide, and exposure is increasing as the climate warms. Despite the large disease burden, risk assessment and risk reduction activities have suffered from limited information regarding exposure-outcome associations for a wide range of pollens, potential confounders of observed associations, and knowledge of exposure thresholds for symptom development in sensitized individuals. Our findings confirm known exposure outcome associations for a number of pollens while controlling for a larger range of potential confounders than prior research and extending the findings to cover much of the contiguous United States and extending the catalogue to several other pollen categories. Our findings highlight the significance of even moderate levels of pollen exposure and have implications for future efforts at estimating disease burden associated with aeroallergen exposure in a warming world and for guiding efforts to minimize harms related to climate change adaptation and mitigation activities.

## Supplementary Information

**Additional file 1.** Figure.

**Additional file 2.** Supplemental information.

## Data Availability

The health data that support the findings of this study are available from IBM Truven, and the pollen data are available through the National Allergy Bureau. These datasets are proprietary and were used under license for the current study. Restrictions apply to the availability of these data, which and so are not publicly available.

## Abbreviations

NAB: National Allergy Bureau
ILI: Influenza-like illness
OTC: Over the counter
ED: Emergency department
AAAAI: American Academy of Allergy, Asthma, and Immunology
MSA: Metropolitan Statistical Area
CCAE: Commercial Claims and Encounters
ICD: International Classification of Diseases
AR: Allergic Rhinitis
MR: Medication refill
CDC: Centers for Disease Control
HHS: Health and Human Services
NLDAS: North American Land Data Assimilation System
RR: Relative risk
