# Supplementary figures and images for: Short term physician visits and medication prescriptions for allergic disease associated with seasonal tree, grass, and weed pollen exposure across the United States
Source: Environ Health. 2021 Jul 21;20:85. doi: 10.1186/s12940-021-00766-3 (PMC8296728; doi:10.1186/s12940-021-00766-3)

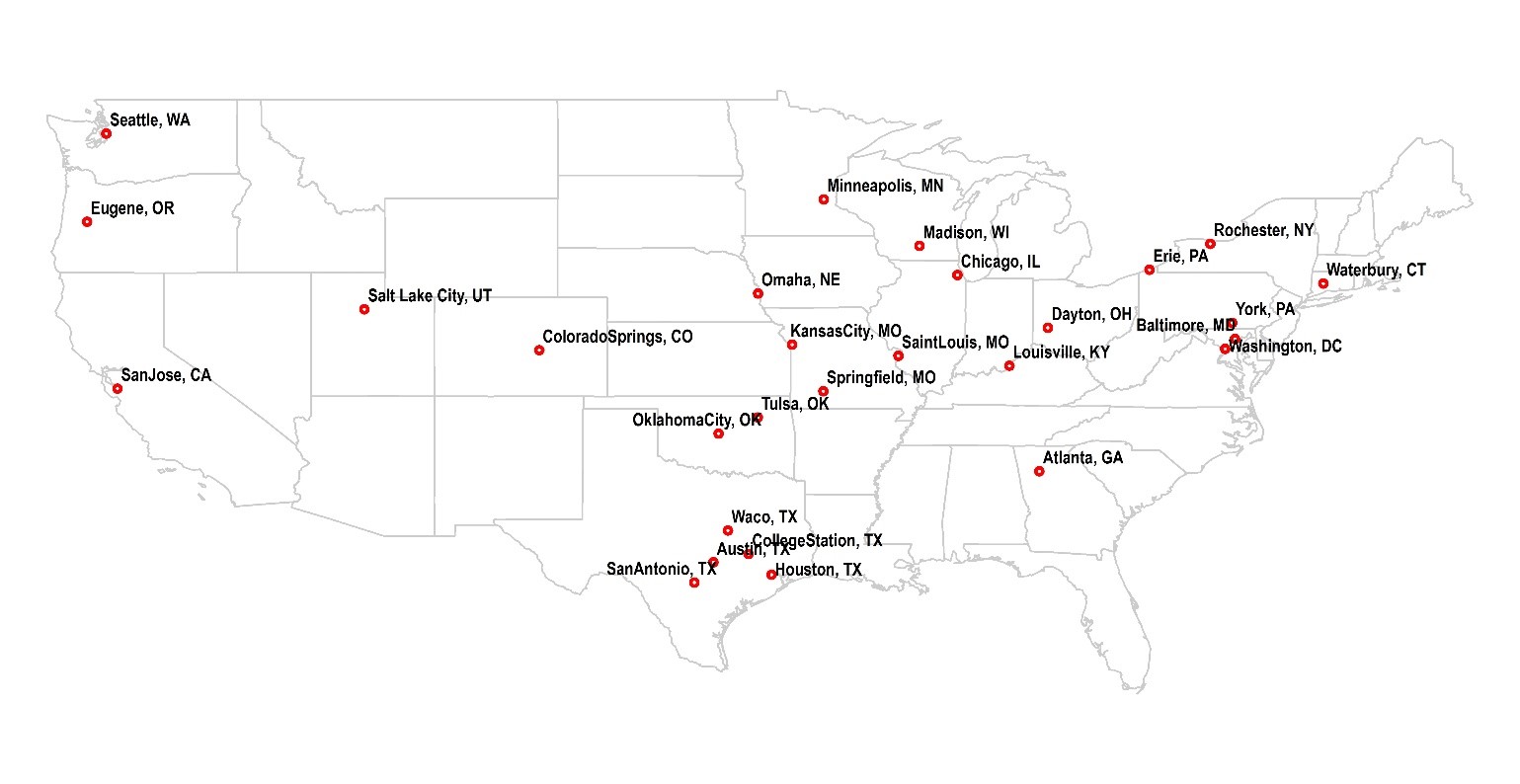

Supplement: Supplementary file 1 — Additional file 1. Figure. [file 12940_2021_766_MOESM1_ESM.jpg]
