# Supplementary material for: Short term physician visits and medication prescriptions for allergic disease associated with seasonal tree, grass, and weed pollen exposure across the United States
Source: Environ Health. 2021 Jul 21;20:85. doi: 10.1186/s12940-021-00766-3 (PMC8296728; doi:10.1186/s12940-021-00766-3)
Supplement: Supplementary file 2 — Additional file 2. Supplemental information. [file 12940_2021_766_MOESM2_ESM.docx]

**SUPPLEMENTAL INFORMATION**


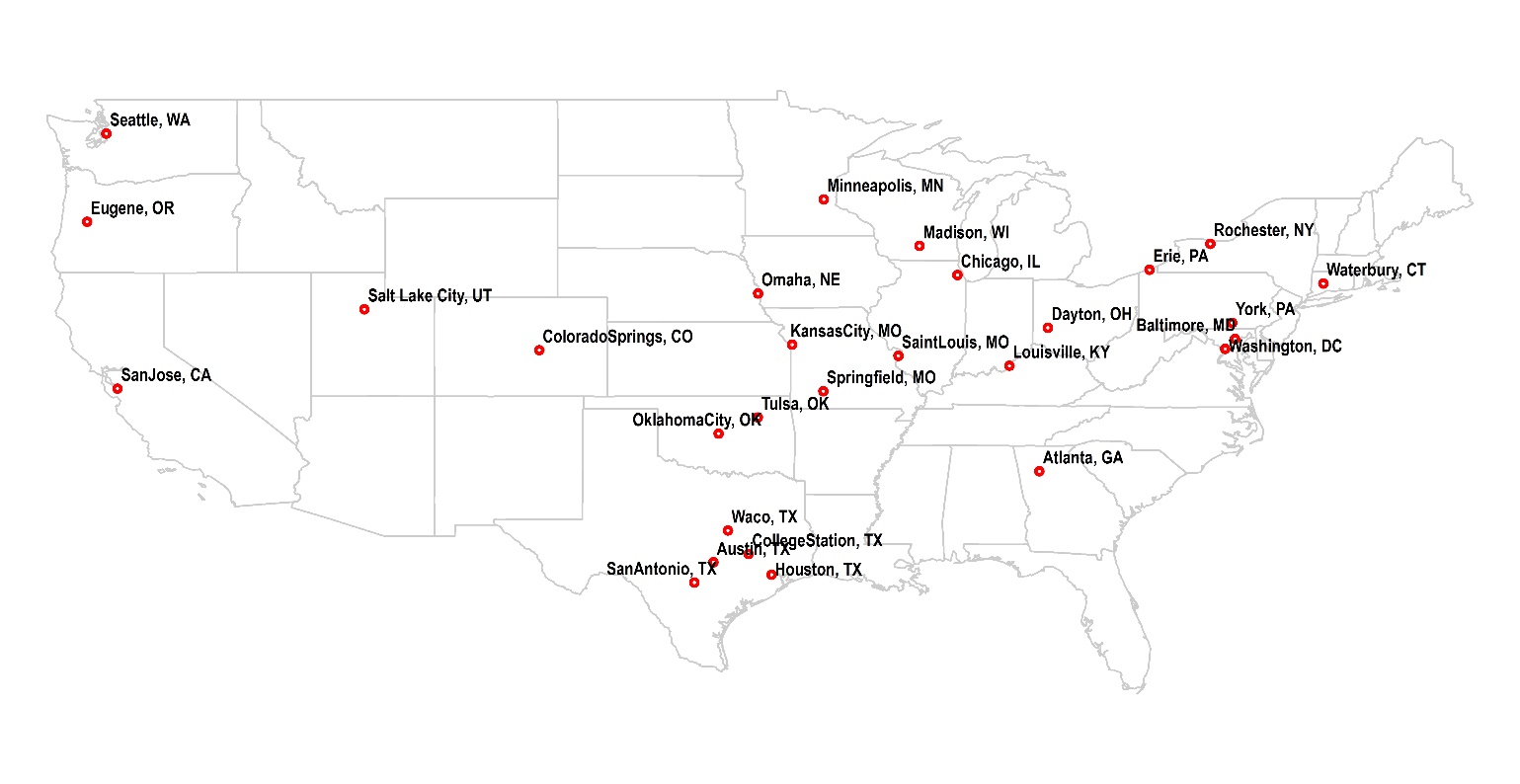


Supplemental figure SF1. Map showing locations of the pollen stations used in the analyses.

|  | Tree | Grass | Weed |
| --- | --- | --- | --- |
| *National Allergy Bureau categories* | | | |
| Low | 0-14 | 0-4 | 0-9 |
| Moderate | 15-89 | 5-19 | 10-49 |
| High | 90-149 | 20-199 | 50-499 |
| Very high | >1500 | >200 | >500 |
| *Modified categories* | | | |
| Low | 0-14 | 0-4 | 0-9 |
| Moderate | 15-89 | 5-19 | 10-49 |
| Moderately high | 90-249 | 20-49 | 50-99 |
| High | 250-1500 | 50-100 | 100-250 |
| Very high | >1500 | >100 | >250 |

Supplemental table ST1. Pollen categories (grain*day/m^3^) based on specified by the National Allergy Bureau and modified categories used in this analysis.

| Type | Generic name | Product name | ATC code |
| --- | --- | --- | --- |
| Antihistamine | Loratidine | Claritin, Alavert | R06AX13 |
| Antihistamine | Diphenhydramine Hydrochloride | Benadryl | R06AA02 |
| Antihistamine | Ceterizine Hydrochloride | Zyrtec | R06AE07 |
| Antihistamine | Fexofenadine | Allegra | R06AX26 |
| Antihistamine | Pseudoephedrine Hydrochloride | Sudafed | R06AB56 |
| Steroid | Mometasone Furoate | Nasonex | R03BA07 |
| Steroid | Fluticasone Propionate | Flonase | R01AD08 |
| Steroid | Triamcinolone Acetonide | Nasacort AQ | H02AB08 |
| Steroid | Budesonide | Rhinocort | R03BA02 |
| Steroid | Fluticasone Furoate | Veramyst | R03BA09 |
| Steroid | Ciclesonide | Omnaris | R03BA08 |
| Antihistamine | Azelastine Hydrochloride | Astepro | R06AX19 |
| Antihistamine | Olopatadine Hydrochloride | Patanase | R01AC08 |
| Antihistamine | Clemastine Fumarate | Tavist | R06AA04 |

Supplemental table ST2. List of prescription allergy medications used in this analysis.

| Location | Allergy medication fills | | | | Allergic rhinitis (first visit) | | | |
| --- | --- | --- | --- | --- | --- | --- | --- | --- |
|  | Female | Male | 0-17 years | 18-65 years | Female | Male | 0-17 years | 18-65 years |
| Atlanta, GA | 548538 | 463130 | 136002 | 875666 | 81112 | 71293 | 38607 | 113798 |
| Austin, TX | 206465 | 194854 | 48698 | 352621 | 47261 | 42852 | 14621 | 75492 |
| Baltimore, MD | 91834 | 87887 | 25008 | 154713 | 27523 | 26873 | 12415 | 41981 |
| Chicago, IL | 406221 | 389184 | 105013 | 690392 | 56803 | 56190 | 31977 | 81016 |
| College Station, TX | 25501 | 24845 | 5952 | 44394 | 4305 | 4365 | 1432 | 7238 |
| Colorado Springs, CO | 15891 | 15591 | 4392 | 27090 | 4805 | 4907 | 2646 | 7066 |
| Dayton, OH | 37473 | 35345 | 8222 | 64596 | 13864 | 13720 | 5381 | 22203 |
| Erie, PA | 10656 | 11179 | 3167 | 18668 | 2800 | 2914 | 1634 | 4080 |
| Eugene, OR | 12978 | 11784 | 1476 | 23286 | 2480 | 2344 | 656 | 4168 |
| Houston, TX | 275047 | 256573 | 79114 | 452506 | 55643 | 54026 | 23353 | 86316 |
| Kansas City, MO | 102535 | 95935 | 29828 | 168642 | 7182 | 7136 | 5014 | 9304 |
| Louisville, KY | 90972 | 85854 | 25822 | 151004 | 27465 | 26977 | 12541 | 41901 |
| Madison, WI | 7752 | 8143 | 2059 | 13836 | 498 | 504 | 301 | 701 |
| Minneapolis, MN | 58829 | 57472 | 14532 | 101769 | 5128 | 5235 | 2682 | 7681 |
| Oklahoma City, OK | 124550 | 116379 | 29150 | 211779 | 21549 | 19112 | 10834 | 29827 |
| Omaha, NE | 24410 | 23845 | 7288 | 40967 | 2966 | 3015 | 1691 | 4290 |
| Rochester, NY | 18754 | 18971 | 4564 | 33161 | 2284 | 2367 | 1384 | 3267 |
| Saint Louis, MO | 209722 | 192719 | 51267 | 351174 | 33057 | 31618 | 16404 | 48271 |
| Salt Lake City, UT | 23429 | 23209 | 4381 | 42257 | 6401 | 6599 | 2825 | 10175 |
| San Antonio, TX | 92932 | 86666 | 30504 | 149094 | 27364 | 23932 | 12821 | 38475 |
| San Jose, CA | 89273 | 87356 | 21145 | 155484 | 12214 | 11575 | 5494 | 18295 |
| Seattle, WA | 124787 | 128380 | 23706 | 229461 | 12280 | 12235 | 5008 | 19507 |
| Springfield, MO | 10084 | 9588 | 2502 | 17170 | 989 | 978 | 494 | 1473 |
| Tulsa, OK | 72432 | 68280 | 18572 | 122140 | 15465 | 15173 | 7384 | 23254 |
| Waco, TX | 42271 | 39289 | 8306 | 73254 | 13416 | 13136 | 6471 | 20081 |
| Washington, DC | 156954 | 149630 | 45451 | 261133 | 49925 | 49537 | 25122 | 74340 |
| Waterbury, CT | 27802 | 26711 | 7250 | 47263 | 9343 | 9456 | 3525 | 15274 |
| York, PA | 16584 | 16595 | 4037 | 29142 | 7494 | 7205 | 3488 | 11211 |

Supplemental table ST3. Location-specific number of patients reporting allergy medications fills and allergic rhinitis physician visit by gender and age groups.

| Location | Pollen levels | Tree pollen | | | Grass pollen | | | Weeds pollen | | |
| --- | --- | --- | --- | --- | --- | --- | --- | --- | --- | --- |
|  |  | Rel Risk | 95% CI | | Rel Risk | 95% CI | | Rel Risk | 95% CI | |
| Atlanta, GA | *Very high* | 1.26 | *1.22* | *1.31* |  |  |  |  |  |  |
|  | *High* | 1.14 | *1.11* | *1.18* |  |  |  |  |  |  |
|  | *Moderately high* | 1.10 | *1.07* | *1.13* |  |  |  |  |  |  |
|  | *Moderate* | 1.04 | *1.02* | *1.06* |  |  |  |  |  |  |
| Austin, TX | *Very high* | 1.16 | *1.13* | *1.20* |  |  |  | 1.19 | *1.09* | *1.28* |
|  | *High* | 1.09 | *1.06* | *1.11* |  |  |  | 1.19 | *1.14* | *1.25* |
|  | *Moderately high* | 1.05 | *1.02* | *1.09* |  |  |  | 1.11 | *1.06* | *1.17* |
|  | *Moderate* | 1.06 | *1.03* | *1.08* |  |  |  | 1.06 | *1.02* | *1.09* |
| Baltimore, MD | *Very high* | 1.40 | *1.33* | *1.47* | 1.05 | *0.98* | *1.12* |  |  |  |
|  | *High* | 1.24 | *1.19* | *1.29* | 0.99 | *0.94* | *1.04* |  |  |  |
|  | *Moderately high* | 1.06 | *1.01* | *1.11* | 0.99 | *0.95* | *1.04* |  |  |  |
|  | *Moderate* | 1.07 | *1.03* | *1.10* | 1.02 | *0.99* | *1.04* |  |  |  |
| Chicago, IL | *Very high* | 1.30 | *1.16* | *1.43* |  |  |  | 1.16 | *0.98* | *1.35* |
|  | *High* | 1.19 | *1.13* | *1.25* |  |  |  | 1.08 | *1.01* | *1.15* |
|  | *Moderately high* | 1.09 | *1.04* | *1.14* |  |  |  | 1.06 | *1.01* | *1.10* |
|  | *Moderate* | 1.06 | *1.02* | *1.10* |  |  |  | 1.00 | *0.97* | *1.02* |
| College Station, TX | *Very high* | 1.15 | *1.06* | *1.24* | 1.23 | *1.06* | *1.40* | 1.23 | *1.14* | *1.31* |
|  | *High* | 1.08 | *1.03* | *1.14* | 0.98 | *0.84* | *1.11* | 1.10 | *1.03* | *1.18* |
|  | *Moderately high* | 1.10 | *1.05* | *1.16* | 1.04 | *0.97* | *1.11* | 1.02 | *0.95* | *1.09* |
|  | *Moderate* | 1.01 | *0.97* | *1.05* | 1.01 | *0.98* | *1.05* | 1.00 | *0.96* | *1.05* |
| Colorado Springs, CO | *Very high* | 0.93 | *0.51* | *1.35* | 1.20 | *0.91* | *1.49* | 1.27 | *1.01* | *1.52* |
|  | *High* | 1.03 | *0.95* | *1.11* | 1.13 | *0.98* | *1.28* | 1.02 | *0.92* | *1.12* |
|  | *Moderately high* | 1.02 | *0.94* | *1.09* | 1.02 | *0.95* | *1.10* | 1.05 | *0.96* | *1.13* |
|  | *Moderate* | 0.99 | *0.93* | *1.05* | 0.98 | *0.94* | *1.02* | 1.01 | *0.96* | *1.05* |
| Dayton, OH | *Very high* | 1.24 | *1.16* | *1.32* | 0.97 | *0.86* | *1.08* | 1.06 | *0.95* | *1.17* |
|  | *High* | 1.10 | *1.04* | *1.16* | 0.94 | *0.86* | *1.03* | 1.09 | *1.03* | *1.16* |
|  | *Moderately high* | 1.09 | *1.04* | *1.15* | 0.99 | *0.93* | *1.04* | 1.12 | *1.05* | *1.18* |
|  | *Moderate* | 1.02 | *0.98* | *1.06* | 0.98 | *0.95* | *1.02* | 0.99 | *0.95* | *1.03* |
| Erie, PA | *Very high* | 1.44 | *1.29* | *1.59* | 0.83 | *0.60* | *1.06* |  |  |  |
|  | *High* | 1.19 | *1.10* | *1.29* | 1.02 | *0.89* | *1.14* | 1.03 | *0.89* | *1.17* |
|  | *Moderately high* | 1.14 | *1.04* | *1.23* | 0.90 | *0.81* | *1.00* | 0.99 | *0.90* | *1.09* |
|  | *Moderate* | 1.08 | *1.00* | *1.16* | 1.00 | *0.95* | *1.05* | 1.02 | *0.97* | *1.08* |
| Eugene, OR | *Very high* | 1.09 | *0.72* | *1.46* | 1.43 | *1.34* | *1.53* |  |  |  |
|  | *High* | 1.11 | *1.01* | *1.20* | 1.27 | *1.16* | *1.37* |  |  |  |
|  | *Moderately high* | 1.12 | *1.05* | *1.18* | 1.10 | *1.01* | *1.19* |  |  |  |
|  | *Moderate* | 1.05 | *1.00* | *1.10* | 1.08 | *1.02* | *1.15* |  |  |  |
| Houston, TX | *Very high* | 1.09 | *1.03* | *1.15* | 1.11 | *0.93* | *1.30* | 1.14 | *1.09* | *1.20* |
|  | *High* | 1.07 | *1.03* | *1.11* | 1.02 | *0.95* | *1.10* | 1.09 | *1.03* | *1.15* |
|  | *Moderately high* | 1.08 | *1.04* | *1.12* | 0.95 | *0.91* | *0.99* | 1.04 | *0.99* | *1.09* |
|  | *Moderate* | 1.01 | *0.99* | *1.04* | 0.98 | *0.96* | *1.00* | 1.00 | *0.96* | *1.04* |
| Kansas City, MO | *Very high* | 1.16 | *1.10* | *1.22* | 1.04 | *0.99* | *1.09* | 1.24 | *1.19* | *1.30* |
|  | *High* | 1.09 | *1.04* | *1.14* | 0.99 | *0.94* | *1.04* | 1.14 | *1.09* | *1.19* |
|  | *Moderately high* | 1.05 | *1.00* | *1.10* | 0.98 | *0.94* | *1.02* | 1.09 | *1.03* | *1.14* |
|  | *Moderate* | 1.05 | *1.01* | *1.09* | 0.98 | *0.95* | *1.01* | 1.00 | *0.97* | *1.03* |
| Louisville, KY | *Very high* | 1.12 | *1.03* | *1.21* | 1.03 | *0.93* | *1.14* | 1.15 | *1.05* | *1.26* |
|  | *High* | 1.06 | *1.02* | *1.10* | 1.01 | *0.94* | *1.08* | 1.11 | *1.05* | *1.16* |
|  | *Moderately high* | 1.01 | *0.96* | *1.05* | 1.07 | *1.02* | *1.11* | 1.14 | *1.09* | *1.19* |
|  | *Moderate* | 1.00 | *0.97* | *1.04* | 1.03 | *1.00* | *1.06* | 1.04 | *1.00* | *1.08* |
| Madison, WI | *Very high* | 1.18 | *0.93* | *1.43* |  |  |  | 1.20 | *0.96* | *1.45* |
|  | *High* | 1.15 | *1.02* | *1.27* |  |  |  | 0.98 | *0.86* | *1.10* |
|  | *Moderately high* | 1.18 | *1.07* | *1.30* |  |  |  | 0.98 | *0.87* | *1.08* |
|  | *Moderate* | 1.07 | *0.96* | *1.17* |  |  |  | 1.04 | *0.97* | *1.11* |
| Minneapolis, MN | *Very high* | 1.20 | *1.11* | *1.29* |  |  |  | 1.22 | *1.08* | *1.36* |
|  | *High* | 1.13 | *1.06* | *1.20* |  |  |  | 1.05 | *0.98* | *1.12* |
|  | *Moderately high* | 1.12 | *1.05* | *1.19* |  |  |  | 1.02 | *0.96* | *1.07* |
|  | *Moderate* | 1.08 | *1.02* | *1.14* |  |  |  | 1.01 | *0.96* | *1.05* |
| Oklahoma City, OK | *Very high* | 1.03 | *0.97* | *1.09* | 0.98 | *0.91* | *1.05* | 1.14 | *1.08* | *1.19* |
|  | *High* | 1.05 | *1.02* | *1.08* | 0.99 | *0.94* | *1.03* | 1.11 | *1.06* | *1.17* |
|  | *Moderately high* | 1.02 | *0.98* | *1.05* | 0.97 | *0.94* | *1.01* | 1.07 | *1.02* | *1.12* |
|  | *Moderate* | 1.00 | *0.98* | *1.03* | 0.97 | *0.94* | *1.00* | 1.03 | *1.00* | *1.06* |
| Omaha, NE | *Very high* | 1.22 | *1.12* | *1.33* | 0.76 | *0.57* | *0.94* | 1.14 | *1.06* | *1.22* |
|  | *High* | 1.13 | *1.06* | *1.20* | 0.97 | *0.85* | *1.08* | 1.07 | *0.99* | *1.14* |
|  | *Moderately high* | 1.04 | *0.97* | *1.10* | 0.91 | *0.84* | *0.97* | 1.06 | *0.99* | *1.13* |
|  | *Moderate* | 1.06 | *1.00* | *1.11* | 0.97 | *0.93* | *1.01* | 1.03 | *0.99* | *1.08* |
| Rochester, NY | *Very high* | 1.70 | *1.37* | *2.03* | 1.10 | *0.96* | *1.24* | 1.04 | *0.92* | *1.16* |
|  | *High* | 1.23 | *1.13* | *1.34* | 1.15 | *1.05* | *1.25* | 1.06 | *0.99* | *1.14* |
|  | *Moderately high* | 1.18 | *1.08* | *1.28* | 1.03 | *0.96* | *1.11* | 1.12 | *1.03* | *1.21* |
|  | *Moderate* | 1.05 | *0.96* | *1.13* | 1.02 | *0.96* | *1.08* | 1.02 | *0.96* | *1.09* |
| Saint Louis, MO | *Very high* | 1.25 | *1.20* | *1.30* | 0.97 | *0.89* | *1.05* | 1.01 | *0.94* | *1.09* |
|  | *High* | 1.11 | *1.08* | *1.15* | 1.04 | *0.97* | *1.10* | 1.02 | *0.98* | *1.06* |
|  | *Moderately high* | 1.06 | *1.02* | *1.10* | 0.98 | *0.94* | *1.02* | 1.01 | *0.97* | *1.05* |
|  | *Moderate* | 1.05 | *1.02* | *1.08* | 0.99 | *0.96* | *1.02* | 0.98 | *0.96* | *1.01* |
| Salt Lake City, UT | *Very high* | 0.91 | *0.58* | *1.25* |  |  |  |  |  |  |
|  | *High* | 1.06 | *0.98* | *1.14* |  |  |  |  |  |  |
|  | *Moderately high* | 1.06 | *1.00* | *1.12* |  |  |  |  |  |  |
|  | *Moderate* | 1.06 | *1.02* | *1.11* |  |  |  |  |  |  |
| San Antonio, TX | *Very high* | 1.06 | *1.00* | *1.13* |  |  |  | 1.16 | *1.09* | *1.23* |
|  | *High* | 1.03 | *0.99* | *1.07* |  |  |  | 1.09 | *1.04* | *1.14* |
|  | *Moderately high* | 1.01 | *0.97* | *1.05* |  |  |  | 1.02 | *0.98* | *1.07* |
|  | *Moderate* | 1.03 | *1.00* | *1.06* |  |  |  | 1.00 | *0.97* | *1.03* |
| San Jose, CA | *Very high* | 1.09 | *0.98* | *1.21* | 1.23 | *1.14* | *1.31* |  |  |  |
|  | *High* | 1.11 | *1.07* | *1.15* | 1.15 | *1.08* | *1.23* |  |  |  |
|  | *Moderately high* | 1.07 | *1.04* | *1.10* | 1.06 | *1.01* | *1.11* |  |  |  |
|  | *Moderate* | 1.04 | *1.01* | *1.06* | 1.03 | *1.00* | *1.06* |  |  |  |
| Seattle, WA | *Very high* | 1.10 | *1.01* | *1.18* |  |  |  |  |  |  |
|  | *High* | 1.07 | *1.03* | *1.11* |  |  |  |  |  |  |
|  | *Moderately high* | 1.03 | *0.99* | *1.06* |  |  |  |  |  |  |
|  | *Moderate* | 1.01 | *0.98* | *1.04* |  |  |  |  |  |  |
| Springfield, MO | *Very high* | 1.14 | *0.95* | *1.34* | 1.11 | *0.97* | *1.24* | 1.21 | *1.07* | *1.34* |
|  | *High* | 1.04 | *0.94* | *1.15* | 0.90 | *0.76* | *1.04* | 1.03 | *0.92* | *1.15* |
|  | *Moderately high* | 1.05 | *0.94* | *1.15* | 0.99 | *0.91* | *1.06* | 1.00 | *0.91* | *1.08* |
|  | *Moderate* | 1.05 | *0.96* | *1.14* | 1.01 | *0.96* | *1.06* | 1.01 | *0.96* | *1.06* |
| Tulsa, OK | *Very high* | 1.12 | *1.06* | *1.18* | 1.06 | *0.98* | *1.15* | 1.19 | *1.13* | *1.24* |
|  | *High* | 1.07 | *1.03* | *1.11* | 1.04 | *0.99* | *1.09* | 1.09 | *1.04* | *1.13* |
|  | *Moderately high* | 1.04 | *0.99* | *1.08* | 1.02 | *0.98* | *1.05* | 1.05 | *1.00* | *1.10* |
|  | *Moderate* | 1.04 | *1.01* | *1.07* | 1.02 | *0.99* | *1.05* | 1.02 | *0.99* | *1.05* |
| Waco, TX | *Very high* | 1.14 | *1.03* | *1.24* | 1.05 | *0.97* | *1.13* | 1.09 | *1.02* | *1.16* |
|  | *High* | 1.10 | *1.05* | *1.15* | 1.01 | *0.93* | *1.08* | 1.02 | *0.96* | *1.09* |
|  | *Moderately high* | 1.07 | *1.02* | *1.11* | 0.97 | *0.91* | *1.02* | 0.99 | *0.92* | *1.06* |
|  | *Moderate* | 1.04 | *1.00* | *1.08* | 0.95 | *0.91* | *0.99* | 0.96 | *0.91* | *1.01* |
| Washington, DC | *Very high* | 1.50 | *1.43* | *1.56* |  |  |  |  |  |  |
|  | *High* | 1.23 | *1.19* | *1.27* |  |  |  |  |  |  |
|  | *Moderately high* | 1.13 | *1.09* | *1.17* |  |  |  |  |  |  |
|  | *Moderate* | 1.07 | *1.04* | *1.11* |  |  |  |  |  |  |
| Waterbury, CT | *Very high* | 1.47 | *1.38* | *1.55* |  |  |  |  |  |  |
|  | *High* | 1.13 | *1.06* | *1.20* |  |  |  |  |  |  |
|  | *Moderately high* | 1.04 | *0.96* | *1.11* |  |  |  |  |  |  |
|  | *Moderate* | 1.05 | *0.99* | *1.12* |  |  |  |  |  |  |
| York, PA | *Very high* | 1.32 | *1.17* | *1.47* | 1.01 | *0.77* | *1.24* |  |  |  |
|  | *High* | 1.19 | *1.11* | *1.27* | 1.00 | *0.91* | *1.09* |  |  |  |
|  | *Moderately high* | 1.08 | *1.00* | *1.16* | 0.98 | *0.90* | *1.05* |  |  |  |
|  | *Moderate* | 1.05 | *0.98* | *1.11* | 1.00 | *0.94* | *1.06* |  |  |  |

Supplemental table ST4. Location-specific relative risk estimates of allergy medication fill for five different categories of tree, grass and weed pollen concentrations using the modified NAB thresholds. Same-day pollen concentrations are used to define the pollen categories. Relative risk estimates derived from models with weekly ILI index, air pollution measures (daily PM2.5 and Ozone) and meteorological factors (daily maximum temperature, total precipitation, and average wind speed) as covariates.

| Location | Pollen levels | Tree pollen | | | Grass pollen | | | Weeds pollen | | |
| --- | --- | --- | --- | --- | --- | --- | --- | --- | --- | --- |
|  |  | Rel Risk | 95% CI | | Rel Risk | 95% CI | | Rel Risk | 95% CI | |
| Atlanta, GA | *Very high* | 1.57 | *1.42* | *1.72* |  |  |  |  |  |  |
|  | *High* | 1.18 | *1.05* | *1.30* |  |  |  |  |  |  |
|  | *Moderately high* | 1.14 | *1.02* | *1.26* |  |  |  |  |  |  |
|  | *Moderate* | 0.99 | *0.90* | *1.09* |  |  |  |  |  |  |
| Austin, TX | *Very high* | 1.05 | *0.92* | *1.19* |  |  |  | 1.56 | *1.13* | *1.98* |
|  | *High* | 0.99 | *0.88* | *1.10* |  |  |  | 1.52 | *1.26* | *1.78* |
|  | *Moderately high* | 1.00 | *0.89* | *1.12* |  |  |  | 1.29 | *1.05* | *1.54* |
|  | *Moderate* | 1.08 | *0.99* | *1.16* |  |  |  | 1.10 | *0.93* | *1.26* |
| Baltimore, MD | *Very high* | 1.01 | *0.82* | *1.20* | 1.66 | *1.46* | *1.86* |  |  |  |
|  | *High* | 1.22 | *1.09* | *1.35* | 1.48 | *1.34* | *1.61* |  |  |  |
|  | *Moderately high* | 0.85 | *0.72* | *0.98* | 1.36 | *1.24* | *1.49* |  |  |  |
|  | *Moderate* | 0.98 | *0.88* | *1.07* | 1.04 | *0.95* | *1.13* |  |  |  |
| Chicago, IL | *Very high* | 1.47 | *1.16* | *1.77* |  |  |  | 1.18 | *0.72* | *1.64* |
|  | *High* | 1.68 | *1.51* | *1.84* |  |  |  | 1.76 | *1.56* | *1.97* |
|  | *Moderately high* | 1.20 | *1.06* | *1.35* |  |  |  | 1.19 | *1.03* | *1.35* |
|  | *Moderate* | 1.19 | *1.06* | *1.32* |  |  |  | 1.09 | *1.01* | *1.18* |
| College Station, TX | *Very high* | 1.69 | *1.50* | *1.88* | 1.05 | *0.51* | *1.60* | 1.01 | *0.62* | *1.39* |
|  | *High* | 1.63 | *1.50* | *1.77* | 0.64 | *0.21* | *1.08* | 1.26 | *0.96* | *1.56* |
|  | *Moderately high* | 0.94 | *0.79* | *1.09* | 1.15 | *0.96* | *1.35* | 1.24 | *0.99* | *1.49* |
|  | *Moderate* | 1.01 | *0.89* | *1.13* | 1.31 | *1.20* | *1.41* | 1.07 | *0.95* | *1.18* |
| Colorado Springs, CO | *Very high* | 0.51 | *-0.11* | *1.13* | 1.45 | *0.12* | *2.78* | 0.96 | *-0.40* | *2.31* |
|  | *High* | 1.09 | *0.91* | *1.27* | 1.88 | *1.38* | *2.38* | 1.32 | *0.91* | *1.72* |
|  | *Moderately high* | 0.84 | *0.66* | *1.01* | 1.12 | *0.87* | *1.36* | 1.44 | *1.12* | *1.77* |
|  | *Moderate* | 0.99 | *0.84* | *1.15* | 1.10 | *0.97* | *1.23* | 1.34 | *1.19* | *1.49* |
| Dayton, OH | *Very high* | 0.89 | *0.67* | *1.10* | 1.24 | *1.00* | *1.49* | 1.25 | *0.85* | *1.64* |
|  | *High* | 1.11 | *0.95* | *1.26* | 1.10 | *0.91* | *1.30* | 1.33 | *1.11* | *1.56* |
|  | *Moderately high* | 1.12 | *0.97* | *1.26* | 1.30 | *1.17* | *1.43* | 1.16 | *0.92* | *1.40* |
|  | *Moderate* | 1.01 | *0.90* | *1.12* | 1.20 | *1.10* | *1.29* | 1.09 | *0.97* | *1.21* |
| Erie, PA | *Very high* | 2.24 | *1.90* | *2.58* | 0.74 | *0.10* | *1.38* |  |  |  |
|  | *High* | 1.74 | *1.53* | *1.95* | 1.22 | *0.93* | *1.51* | 0.67 | *0.13* | *1.20* |
|  | *Moderately high* | 1.57 | *1.35* | *1.78* | 1.57 | *1.35* | *1.78* | 0.89 | *0.52* | *1.26* |
|  | *Moderate* | 1.39 | *1.22* | *1.57* | 1.15 | *1.00* | *1.30* | 0.83 | *0.68* | *0.99* |
| Eugene, OR | *Very high* | 2.26 | *1.36* | *3.16* | 1.80 | *1.59* | *2.01* |  |  |  |
|  | *High* | 1.15 | *0.94* | *1.36* | 1.44 | *1.22* | *1.67* |  |  |  |
|  | *Moderately high* | 1.16 | *1.00* | *1.31* | 1.40 | *1.19* | *1.60* |  |  |  |
|  | *Moderate* | 1.12 | *1.01* | *1.22* | 1.46 | *1.32* | *1.61* |  |  |  |
| Houston, TX | *Very high* | 1.26 | *1.07* | *1.45* | 0.94 | *0.14* | *1.74* | 1.47 | *1.24* | *1.71* |
|  | *High* | 1.24 | *1.09* | *1.38* | 1.48 | *1.22* | *1.74* | 1.28 | *1.03* | *1.52* |
|  | *Moderately high* | 0.95 | *0.81* | *1.09* | 0.90 | *0.75* | *1.05* | 1.22 | *1.03* | *1.42* |
|  | *Moderate* | 0.90 | *0.79* | *1.00* | 0.94 | *0.86* | *1.02* | 1.09 | *0.96* | *1.23* |
| Kansas City, MO | *Very high* | 1.34 | *1.17* | *1.52* | 1.40 | *1.25* | *1.55* | 1.46 | *1.28* | *1.64* |
|  | *High* | 1.02 | *0.87* | *1.17* | 1.43 | *1.28* | *1.57* | 1.22 | *1.05* | *1.40* |
|  | *Moderately high* | 0.98 | *0.83* | *1.12* | 1.18 | *1.07* | *1.29* | 1.14 | *0.96* | *1.32* |
|  | *Moderate* | 1.10 | *0.98* | *1.21* | 1.10 | *1.01* | *1.19* | 1.06 | *0.96* | *1.16* |
| Louisville, KY | *Very high* | 1.55 | *1.13* | *1.96* | 0.62 | *0.07* | *1.18* | 0.78 | *0.18* | *1.39* |
|  | *High* | 1.21 | *1.03* | *1.40* | 0.61 | *0.29* | *0.93* | 0.74 | *0.44* | *1.04* |
|  | *Moderately high* | 1.17 | *0.97* | *1.36* | 0.85 | *0.65* | *1.04* | 0.67 | *0.41* | *0.94* |
|  | *Moderate* | 1.03 | *0.85* | *1.20* | 0.85 | *0.71* | *0.99* | 0.80 | *0.61* | *1.00* |
| Madison, WI | *Very high* | 1.28 | *0.76* | *1.80* |  |  |  |  |  |  |
|  | *High* | 0.96 | *0.64* | *1.28* |  |  |  | 1.16 | *0.68* | *1.64* |
|  | *Moderately high* | 0.94 | *0.64* | *1.24* |  |  |  | 1.01 | *0.61* | *1.40* |
|  | *Moderate* | 0.93 | *0.67* | *1.20* |  |  |  | 1.03 | *0.80* | *1.26* |
| Minneapolis, MN | *Very high* | 0.77 | *0.46* | *1.08* |  |  |  | 0.87 | *0.21* | *1.54* |
|  | *High* | 0.95 | *0.74* | *1.17* |  |  |  | 1.06 | *0.73* | *1.38* |
|  | *Moderately high* | 0.97 | *0.75* | *1.18* |  |  |  | 0.89 | *0.66* | *1.12* |
|  | *Moderate* | 0.91 | *0.73* | *1.10* |  |  |  | 0.92 | *0.76* | *1.08* |
| Oklahoma City, OK | *Very high* | 1.30 | *1.11* | *1.49* | 1.24 | *0.98* | *1.50* | 1.41 | *1.16* | *1.66* |
|  | *High* | 0.91 | *0.78* | *1.03* | 1.35 | *1.18* | *1.52* | 1.35 | *1.13* | *1.58* |
|  | *Moderately high* | 0.86 | *0.73* | *0.99* | 1.12 | *0.99* | *1.25* | 1.37 | *1.13* | *1.62* |
|  | *Moderate* | 0.88 | *0.78* | *0.99* | 1.00 | *0.89* | *1.11* | 1.16 | *1.04* | *1.28* |
| Omaha, NE | *Very high* | 1.13 | *0.86* | *1.39* | 1.78 | *1.41* | *2.15* | 1.04 | *0.75* | *1.32* |
|  | *High* | 1.16 | *0.98* | *1.34* | 1.30 | *1.03* | *1.57* | 1.10 | *0.84* | *1.37* |
|  | *Moderately high* | 1.36 | *1.19* | *1.52* | 1.75 | *1.58* | *1.92* | 1.03 | *0.78* | *1.28* |
|  | *Moderate* | 1.25 | *1.11* | *1.40* | 1.21 | *1.09* | *1.33* | 0.91 | *0.77* | *1.06* |
| Rochester, NY | *Very high* | 0.22 | *-1.42* | *1.86* | 1.74 | *1.39* | *2.09* | 0.89 | *0.48* | *1.29* |
|  | *High* | 0.90 | *0.66* | *1.15* | 1.81 | *1.56* | *2.07* | 1.10 | *0.86* | *1.35* |
|  | *Moderately high* | 0.96 | *0.75* | *1.17* | 2.07 | *1.87* | *2.27* | 0.94 | *0.65* | *1.23* |
|  | *Moderate* | 0.99 | *0.80* | *1.17* | 1.78 | *1.61* | *1.96* | 0.98 | *0.82* | *1.15* |
| Saint Louis, MO | *Very high* | 2.03 | *1.81* | *2.24* | 1.44 | *1.15* | *1.73* | 1.00 | *0.44* | *1.56* |
|  | *High* | 1.66 | *1.48* | *1.83* | 1.44 | *1.20* | *1.67* | 0.95 | *0.68* | *1.23* |
|  | *Moderately high* | 1.12 | *0.94* | *1.29* | 1.16 | *1.01* | *1.32* | 0.96 | *0.74* | *1.18* |
|  | *Moderate* | 1.32 | *1.18* | *1.46* | 1.19 | *1.07* | *1.31* | 1.10 | *0.98* | *1.22* |
| Salt Lake City, UT | *Very high* | 0.84 | *0.01* | *1.68* |  |  |  |  |  |  |
|  | *High* | 1.06 | *0.83* | *1.28* |  |  |  |  |  |  |
|  | *Moderately high* | 1.11 | *0.94* | *1.27* |  |  |  |  |  |  |
|  | *Moderate* | 0.97 | *0.84* | *1.09* |  |  |  |  |  |  |
| San Antonio, TX | *Very high* | 0.85 | *0.70* | *1.01* |  |  |  | 1.36 | *1.14* | *1.58* |
|  | *High* | 0.94 | *0.84* | *1.04* |  |  |  | 1.26 | *1.13* | *1.39* |
|  | *Moderately high* | 0.89 | *0.78* | *0.99* |  |  |  | 1.11 | *0.99* | *1.22* |
|  | *Moderate* | 0.93 | *0.86* | *1.00* |  |  |  | 1.05 | *0.98* | *1.12* |
| San Jose, CA | *Very high* | 1.90 | *1.55* | *2.25* | 1.48 | *1.13* | *1.83* |  |  |  |
|  | *High* | 1.40 | *1.27* | *1.52* | 1.07 | *0.82* | *1.33* |  |  |  |
|  | *Moderately high* | 1.06 | *0.95* | *1.17* | 1.06 | *0.90* | *1.22* |  |  |  |
|  | *Moderate* | 1.13 | *1.04* | *1.22* | 1.06 | *0.96* | *1.16* |  |  |  |
| Seattle, WA | *Very high* | 0.94 | *0.67* | *1.21* |  |  |  |  |  |  |
|  | *High* | 0.99 | *0.85* | *1.13* |  |  |  |  |  |  |
|  | *Moderately high* | 1.12 | *1.01* | *1.24* |  |  |  |  |  |  |
|  | *Moderate* | 1.06 | *0.97* | *1.16* |  |  |  |  |  |  |
| Springfield, MO | *Very high* | 2.20 | *1.80* | *2.60* | 0.96 | *0.56* | *1.37* | 0.93 | *0.42* | *1.45* |
|  | *High* | 1.26 | *1.01* | *1.52* | 0.85 | *0.44* | *1.26* | 1.55 | *1.08* | *2.02* |
|  | *Moderately high* | 1.28 | *1.03* | *1.53* | 1.15 | *0.92* | *1.38* | 1.22 | *0.95* | *1.49* |
|  | *Moderate* | 1.26 | *1.03* | *1.49* | 1.06 | *0.89* | *1.22* | 1.17 | *1.01* | *1.33* |
| Tulsa, OK | *Very high* | 1.13 | *0.94* | *1.31* | 0.81 | *0.53* | *1.09* | 1.92 | *1.66* | *2.18* |
|  | *High* | 1.31 | *1.16* | *1.46* | 0.98 | *0.79* | *1.17* | 1.90 | *1.70* | *2.11* |
|  | *Moderately high* | 1.22 | *1.08* | *1.36* | 0.92 | *0.79* | *1.06* | 1.77 | *1.58* | *1.97* |
|  | *Moderate* | 1.05 | *0.93* | *1.17* | 1.06 | *0.95* | *1.16* | 1.34 | *1.23* | *1.45* |
| Waco, TX | *Very high* | 0.77 | *0.44* | *1.11* | 1.20 | *0.93* | *1.47* | 1.97 | *1.65* | *2.29* |
|  | *High* | 0.90 | *0.74* | *1.06* | 1.43 | *1.17* | *1.70* | 2.06 | *1.78* | *2.34* |
|  | *Moderately high* | 1.06 | *0.89* | *1.23* | 1.38 | *1.20* | *1.56* | 1.59 | *1.28* | *1.89* |
|  | *Moderate* | 0.72 | *0.55* | *0.90* | 1.35 | *1.20* | *1.49* | 1.97 | *1.80* | *2.14* |
| Washington, DC | *Very high* | 3.74 | *3.51* | *3.96* |  |  |  |  |  |  |
|  | *High* | 1.60 | *1.45* | *1.75* |  |  |  |  |  |  |
|  | *Moderately high* | 1.34 | *1.19* | *1.48* |  |  |  |  |  |  |
|  | *Moderate* | 1.19 | *1.06* | *1.32* |  |  |  |  |  |  |
| Waterbury, CT | *Very high* | 1.84 | *1.58* | *2.09* |  |  |  |  |  |  |
|  | *High* | 1.62 | *1.42* | *1.81* |  |  |  |  |  |  |
|  | *Moderately high* | 1.41 | *1.20* | *1.62* |  |  |  |  |  |  |
|  | *Moderate* | 1.62 | *1.44* | *1.79* |  |  |  |  |  |  |
| York, PA | *Very high* | 0.69 | *0.22* | *1.15* | 2.25 | *1.66* | *2.85* |  |  |  |
|  | *High* | 0.82 | *0.62* | *1.02* | 1.56 | *1.34* | *1.78* |  |  |  |
|  | *Moderately high* | 0.84 | *0.64* | *1.05* | 1.64 | *1.47* | *1.81* |  |  |  |
|  | *Moderate* | 1.07 | *0.93* | *1.22* | 1.41 | *1.27* | *1.56* |  |  |  |

Supplemental table ST5. Location-specific relative risk estimates of relative risk of physician visits for Allergic Rhinitis (first visit in calendar year) for five different categories of tree, grass and weed pollen concentrations using the modified NAB thresholds. Same-day pollen concentrations are used to define the pollen categories. Relative risk estimates derived from models with weekly ILI index, air pollution measures (daily PM2.5 and Ozone) and meteorological factors (daily maximum temperature, total precipitation, and average wind speed) as covariates.

| **Allergy medication fills (Antihistamine)** | | | | | | | | | |
| --- | --- | --- | --- | --- | --- | --- | --- | --- | --- |
|  | Tree pollen | | | Grass pollen | | | Weed pollen | | |
| **Pollen level** | RR | 95% CI | | RR | 95% CI | | RR | 95% CI | |
| Low | 1 |  |  | 1 |  |  | 1 |  |  |
| Moderate | **1.04** | ***1.03*** | ***1.06*** | 1.00 | *0.98* | *1.03* | 1.00 | *0.97* | *1.04* |
| Moderately high | **1.08** | ***1.05*** | ***1.10*** | 0.99 | *0.97* | *1.01* | **1.03** | ***1.01*** | ***1.06*** |
| High | **1.13** | ***1.09*** | ***1.15*** | **1.04** | ***1.01*** | ***1.08*** | **1.05** | ***1.02*** | ***1.09*** |
| Very high | **1.22** | ***1.15*** | ***1.29*** | 1.06 | *0.98* | *1.13* | **1.11** | ***1.06*** | ***1.17*** |
|  |  |  |  |  |  |  |  |  |  |
| **Allergy medication fills (Steroids)** | | | | | | | | | |
|  | Tree pollen | | | Grass pollen | | | Weed pollen | | |
| **Pollen level** | RR | 95% CI | | RR | 95% CI | | RR | 95% CI | |
| Low | 1 |  |  | 1 |  |  | 1 |  |  |
| Moderate | **1.04** | ***1.02*** | ***1.06*** | 0.99 | *0.97* | *1.01* | **1.02** | ***1.00*** | ***1.04*** |
| Moderately high | **1.06** | ***1.04*** | ***1.08*** | 0.99 | *0.97* | *1.02* | **1.06** | ***1.03*** | ***1.08*** |
| High | **1.09** | ***1.05*** | ***1.13*** | 1.03 | *0.98* | *1.06* | **1.11** | ***1.06*** | ***1.15*** |
| Very high | **1.21** | ***1.15*** | ***1.27*** | 1.07 | *0.99* | *1.14* | **1.17** | ***1.13*** | ***1.21*** |

Supplemental table ST6. Meta-analyzed relative risk estimates of allergy medication fills (separated by antihistamines and steroids) on days with different pollen categories for tree, grass and weed pollen. Same-day pollen concentrations are used to define the pollen categories. Relative risk estimates derived from models with weekly ILI index, air pollution measures (daily PM2.5 and Ozone) and meteorological factors (daily maximum temperature, total precipitation, and average wind speed) as covariates.
